# Supplementary material for: High-quality carnivoran genomes from roadkill samples enable comparative species delineation in aardwolf and bat-eared fox
Source: eLife. 2021 Feb 18;10:e63167. doi: 10.7554/eLife.63167 (PMC7963486; doi:10.7554/eLife.63167)
Supplement: Supplementary file 7. [file elife-63167-supp7.docx]

**Supplementary File 6:** Skull measurements of Proteles taxa from museum specimens and the literature (Allen 1909, Heller 1913 Hollister 1918, Roberts 1932, 1951)

| Specimen | Taxon | Sex | Condylobasal length | Zygomatic width | Greatest brain case width | Inter-orbital breadth | Post-orbital breadth | Mandible length | Reference |
| --- | --- | --- | --- | --- | --- | --- | --- | --- | --- |
| NHMUK ZD 1904.3.1.58 | *P. c. cristatus* | NA | 134 | 77.4 | 44.7 | 24.8 | 29.3 | 93 | Cabrera 1910 |
| NHMUK ZD 1902.9.1.28 | *P. c. cristatus* | NA | 139.1 | 81.3 | 46.3 | 29.4 | 33 | 97 | Cabrera 1910 |
| NHMUK ZD 1934.11.1.5 | *P. c. cristatus* | NA | 134.3 | 77.2 | 44.9 | 24.6 | 26.7 | NA | NHUMUK |
| NHMUK ZD 1883.11.91 | *P. c. cristatus* | NA | 134.5 | 78.3 | 46.4 | 28.4 | 32.2 | NA | NHUMUK |
| MVZ 118478 | *P. c. cristatus* | NA | 126.3 | NA | 46.1 | 23.7 | 31.4 | NA | MVZ |
| MVZ 117841 | *P. c. cristatus* | NA | 132.9 | 81.2 | 46.5 | 25.3 | 27.7 | NA | MVZ |
| Albany District | *P. c. cristatus* | M | 143 | 81.7 | 50 | 28 | 29.5 | 96.5 | Roberts 1951 |
| Albany District | *P. c. cristatus* | M | 135 | 78.5 | 46 | 30.6 | 34.5 | 93 | Roberts 1951 |
| Vryburg | *P. c. cristatus* | M | 136 | 81.5 | 46.5 | 25.4 | 28 | 93 | Roberts 1951 |
| Okanhandja District | *P. c. cristatus* | M | 140 | 84 | 51 | 28.6 | 31.3 | 95 | Roberts 1951 |
| TM 1915 | *P. c. cristatus* | F | 137.5 | 82.2 | 50.5 | 28.2 | 32 | 94.5 | Roberts 1932, 1951; type *transvaalensis* |
| NMS.Z.2020.44 | *P. c. cristatus* | NA | 143.5 | NA | 51.1 | 30.1 | 33.6 | NA | NMS/Twycross Zoo |
| NMS.Z.2020.46.3 | *P. c. cristatus* | NA | 139.1 | NA | 46.2 | 29.5 | 30.7 | NA | NMS/Hamerton Zoo |
| NMS.Z.2020.46.1 | *P. c. cristatus* | NA | 137.4 | NA | 46.6 | 25.6 | 27.2 | NA | NMS/Hamerton Zoo |
| NMS.Z.2020.46.5 | *P. c. cristatus* | NA | 129.2 | NA | 46.4 | 28.2 | 33.9 | NA | NMS/Hamerton Zoo |
| NMS.Z.2020.46.4 | *P. c. cristatus* | NA | 138 | NA | 47.2 | 33.4 | 34.5 | NA | NMS/Hamerton Zoo |
| NHMUK ZD 1904.8.2.25 | *P. c. septentrionalis* | M | 130 | 80 | 45.8 | 36 | 37 | 93 | Cabrera 1910; type *pallidior* |
| NHMUK ZD 1905.12.2.2 | *P. c. septentrionalis* | NA | 134 | 82 | 45 | 31 | 36 | 94 | Cabrera 1910 |
| NHMUK ZD 1895.5.2.2 | *P. c. septentrionalis* | NA | 144 | 85 | 47 | 33.5 | 37 | 102 | Cabrera 1910 |
| USNM 181523 | *P. c. septentrionalis* | F | 136 | 75 | 46 | 26 | 33 | 96 | Heller 1913; type *termes* |
| AMNH 27768 | *P. c. septentrionalis* | M | 141 | 82 | NA | NA | 34 | NA | Allen 1909 |
| USNM 164503 | *P. c. septentrionalis* | M | 140 | 82 | NA | NA | 35.8 | 98 | Hollister 1918 |
| USNM 181495 | *P. c. septentrionalis* | M | 127 | 72 | NA | NA | 33.5 | 89 | Hollister 1918 |
| USNM 164837 | *P. c. septentrionalis* | F | 135 | 88 | NA | NA | 34.8 | 97 | Hollister 1918 |
