# Supplementary material for: High-quality carnivoran genomes from roadkill samples enable comparative species delineation in aardwolf and bat-eared fox
Source: eLife. 2021 Feb 18;10:e63167. doi: 10.7554/eLife.63167 (PMC7963486; doi:10.7554/eLife.63167)
Supplement: Supplementary file 8. [file elife-63167-supp8.docx]

**Table S6.** Sample details and assembly statistics of the 13 newly assembled carnivoran mitochondrial genomes.

| **Species** | **Common name** | **Collection number** | **Origin** | **Sample type** | **Raw reads** | **Mito reads** | **Mean coverage** | **SRA Accession** | **Genbank Accession** |
| --- | --- | --- | --- | --- | --- | --- | --- | --- | --- |
| *Proteles cristatus* | Southern aardwolf | NMB 12641 | South Africa | Tissue | 4,779,321 | 6,665 | 39x | SRR13177413 | Pending |
| *Proteles cristatus* | Southern aardwolf | NMB 12667 | South Africa | Tissue | 9,718,442 | 6,637 | 58x | SRR13177419 | Pending |
| *Proteles cristatus* | Southern aardwolf | FMNH 196086 | Zoo | Tissue | 1,650,154 | 22,600 | 165x | SRR6053051* | Pending |
| *Proteles septentrionalis* | Eastern aardwolf | NMS Z.2018.54 | Tanzania | Tissue | 7,597,504 | 2,709 | 19x | SRR13177412 | Pending |
| *Otocyon megalotis megalotis* | Southern bat-eared fox | NMB 12639 | South Africa | Tissue | 6,719,302 | 4,559 | 27x | SRR13177424 | Pending |
| *Otocyon megalotis megalotis* | Southern bat-eared fox | NMB 12640 | South Africa | Tissue | 9,854,428 | 7,031 | 62x | SRR13177425 | Pending |
| *Otocyon megalotis virgatus* | Eastern bat-eared fox | FMNH 158128 | Tanzania | Tissue | 6,838,309 | 37,719 | 219x | SRR13177414 | Pending |
| *Speothos venaticus* | Bush dog | MNHN 1999-1073 | French Guiana | Tissue | 5,271,782 | 4,763 | 27x | SRR13177428 | Pending |
| *Vulpes vulpes* | Red fox | ISEM T3611 | France | Tissue | 8,355,398 | 3,050 | 18x | SRR13177427 | Pending |
| *Parahyaena brunnea* | Brown hyaena | ISEM FD126 | Toulon Zoo | Feces | 11,739,905 | 3,243 | 17x | SRR13177411 | Pending |
| *Bdeogale nigripes* | Black-footed mongoose | FMNH 167685 | Gabon | Tissue | 6,839,178 | 8,215 | 61x | SRR6053065* | Pending |
| *Fossa fossana* | Malagasy civet | FMNH 156648 | Madagascar | Tissue | 4,914,258 | 17,689 | 131x | SRR6053060* | Pending |
| *Viverra tangalunga* | Malayan civet | FMNH 146957 | Philippines | Tissue | 5,648,442 | 23,116 | 205x | SRR6053069* | Pending |

*: UCE capture reads from Esselstyn et al. (2017); NMB: National Museum, Bloemfontein, South Africa; ISEM: Institut des Sciences de l'Evolution, Montpellier, France; FMNH: Field Museum of Natural History, Chicago, IL, USA; MNHN: Muséum National d’Histoire Naturelle, Paris, France.
